# A Practical Treatise on the Diseases of the Throat and Great Vessels, Including the Principles of Physical Diagnosis

**Published:** 1862-08

**Authors:** 


A Practical Treatise on the Diseases of the Throat and Great Vessels, Including the Principles of Physical Diagnosis. By Walter Hayle Walshe, M.D., Fellow of the Royal College of Physicians; Professor of the Principles and Practice of Medicine, and of Clinical Medicine, University College, London; Physician to University College Hospital; Consulting Physician to the Hospital for Consumption. A new American, from the Third Revised and much Enlarged London Edition. Philadelphia: Blanchard & Lea. 1862.

The former editions of this work have been long enough before the Profession, for their merits to be fully appreciated. The present edition has been thoroughly revised by the author, and is much enlarged, containing 420 royal octavo pages. It is undoubtedly the best work on the diseases of the throat and great vessels, which is accessible to the profession.

For sale by W. B. Keen & Co., Lake Street, Chicago.

